# Association between tea consumption and stroke in the American adult females: analyses of NHANES 2011–2018 data

**DOI:** 10.3389/fnut.2024.1452137

**Published:** 2024-10-22

**Authors:** Yongyue Miao, Sijia Ma, Xian Wu

**Affiliations:** ^1^Graduate School, Heilongjiang University of Chinese Medicine, Harbin, Heilongjiang, China; ^2^Department of Acupuncture and Moxibustion, First Affiliated Hospital of Heilongjiang University of Chinese Medicine, Harbin, Heilongjiang, China

**Keywords:** tea, stroke, female, adult, NHANES

## Abstract

**Background:**

Epidemiological surveys show that there is a significant gender difference in the incidence of stroke, with females having a noticeably higher rate than males. Accordingly, it is crucial to seek preventive measures for stroke specifically targeted at females. Although previous studies have shown that tea has been proven to be negatively correlated with stroke, the relationship between tea and stroke in American adult females is still unclear. Therefore, we aimed to investigate the relationship between tea consumption and the occurrence of stroke in American adult females.

**Methods:**

The data analyzed is derived from the NHANES database between 2011 and 2018. The quantity of tea consumed was gathered from a 24-h dietary review. Stroke was identified by using questionnaire. The association between tea consumption and stroke was investigated using a weighted regression model. Then we used interaction testing and subgroup analysis to conduct a thorough analysis. Simultaneously, the association between the sugar content in tea and stroke was examined.

**Results:**

This study included 5731 adult females aged between 20 and 60 years. Compared to those who did not consume tea, the likelihood of stroke decreased by 9% for each additional 100 g of tea ingested by participants (OR = 0.91, 95%CI: 0.83–1.00). In the unadjusted model, those who drank 307.5–480 g of tea per day had a substantially decreased risk of stroke than those who did not drink tea (OR = 0.23, 95%CI: 0.08–0.64). After adjustment, this relationship also persisted (Model II: OR = 0.23, 95% CI: 0.08–0.64; Model III: OR = 0.23, 95% CI: 0.08–0.66). In both Model II and Model III, there was a statistically significant relationship between consuming 480–744 g of tea per day and the risk of stroke (Model II: OR = 0.39, 95%CI: 0.16–0.94; Model III: OR = 0.42, 95% CI: 0.18–0.98). Subgroup analysis revealed an interaction only with level of education (*P* = 0.031). Ultimately, we also demonstrated that people who drink sugar free tea have a lower risk of stroke, and even after adjusting for mixed factors.

**Conclusion:**

This study suggested that proper tea consumption was associated with a lower risk of stroke in adult females, which recommended drinking sugar free tea.

## 1 Introduction

Stroke is one of the most significant diseases that pose threats to health and life, as well as being the second leading cause of death worldwide (1). According to statistics, in 2019 there were 1,01 million cases of stroke epidemics (2), and incidence increased by 70% in the decade (3). Relevant data shows that about half of stroke survivors have residual effects such as hemiplegia, dysphagia, and dysarthria (4), imposing a significant burden on patients, families, and society. According to Heart Disease and Stroke Statistics(2024) (5), approximately 795,000 new or recurrent strokes occur every year in the United States, with an average death rate of one stroke every 3 min and 14 s, at a cost of up to 56.2 billion dollars, directly or indirectly, and the cost will increase to $943 million by 2035. Therefore, preventing strokes is now more urgent than ever. Primary prevention, which includes effective control of underlying diseases and adoption of a healthy lifestyle, is an effective method to prevent the occurrence of stroke (6–10). By changing lifestyle habits and intervening early on the risk factors for stroke, it is possible to effectively reduce its incidence. Dietary changes are one of these factors, directly affecting the risk of stroke (11, 12) or altering the high-risk factors for stroke (13, 14), recently receiving widespread attention. It is testified that healthy eating is associated with a negative risk of stroke in the numerous cross-sectional and cohort studies (15, 16). Tea is one of the simplest and most healthy beverages that can replace artificially sweetened beverages, with good prospects for stroke prevention and as part of a healthy diet (17).

Tea is the oldest beverage in the world and remains popular globally today (18). The tea contains polyphenols, caffeine, and l-theanine with anti-inflammatory, antioxidant, and neuroprotective effects, which can prevent many diseases, such as cancer, cardiovascular disease, diabetes, stroke and obesity (19, 20). Related investigations have indicated that there was a link between tea consumption and stroke. Based on a prospective cohort study of 500,000 participants, it was discovered that consuming large quantities of tea was linked to a reduced risk of stroke (21). Multiple meta-analyses (22–24) showed the same conclusion that drinking tea was negatively correlated with stroke risk. However, some research revealed the contrary. An epidemiological study revealed no conclusive evidence linking tea consumption to a reduced likelihood of stroke (25). Additionally, a meta-analysis based on 10 cohort studies and 7 case-control studies found that the relationship between tea consumption and stroke risk varies across different regions. With increased tea consumption, the risk of stroke in Australia was found to be increased, whereas in European countries, the risk was found to be decreased (26). In summary, it is necessary to further clarify the association between tea consumption and stroke. Besides the majority of these studies were concentrated on countries with long-established tea consumption habits, such as China, the United Kingdom and Japan, with few studies targeting the American population.

It is worth noting that the average age of stroke onset is steadily decreasing (27), with a significant increase in the incidence of stroke among middle-aged and younger individuals (28). Additionally, stroke ranks as the fifth leading cause among individuals aged 15 to 59 years old (29) according to the World Health Organization’s global burden of diseases (GBD). Given the high rate of disability caused by stroke, which imposes a significant burden on both society and families, it is recommended that we take early action to prevent stroke. As a result, preventive strategies directed primarily at adults are required, however there are few studies in the extant literature. Furthermore, according to the Global stroke statistics 2022 (30), females had more strokes than males (6.4 million /5.8 million), based on GBD stroke statistics from 1990 to 2019. Multiple epidemiological studies have found that females are more prone to stroke than males (31–33), and the same difference exists in mortality (5). According to data from the CDC WONDER study from 2021, more than half of all stroke deaths in the US occur in females (34). In addition, it is reported that the NIHSS score of females after stroke is higher than that of males (35), and they are more likely to have sequelae than males (36). Compared to males, females must experience menstruation, pregnancy, and other unique physiological processes throughout their lives. As a result, females may have more risk factors for stroke than males, such as an excessive number of childbirths and early menopause (37, 38). However, existing research on stroke prevention for patients of a single gender often focuses on males (39). Thus, it is critical for public health to develop a stroke prevention program for adult females.

As previously stated, tea does have a positive impact on health, and it offers even more benefits for females. Some studies have found that tea can prevent premature menopause (40) and can also increase concentrations estradiol in females of childbearing age (41), both of which can affect the occurrence of stroke. Additionally, a randomized controlled trial found that polyphenols (the main component of tea) more effectively reduce the risk of death in females (HR for females:0.42, HR for males:0.76) (42). As a result, it is possible that drinking tea can help adult females avoid stroke. So, the purpose of this study is to investigate the association between adult females’ tea consumption and stroke in order to establish precise recommendations for stroke prevention in real life. We analyzed a nationally representative sample of female adults aged 20–60 years (According to the age classification by WHO, individuals aged 10–19 (adolescents) and those over 60 years old (older adults) have been excluded) from the National Health and Nutrition Examination Survey (NHANES) to investigate the associations of tea consumption with stroke risk.

## 2 Materials and methods

### 2.1 Study population

**FIGURE 1:**
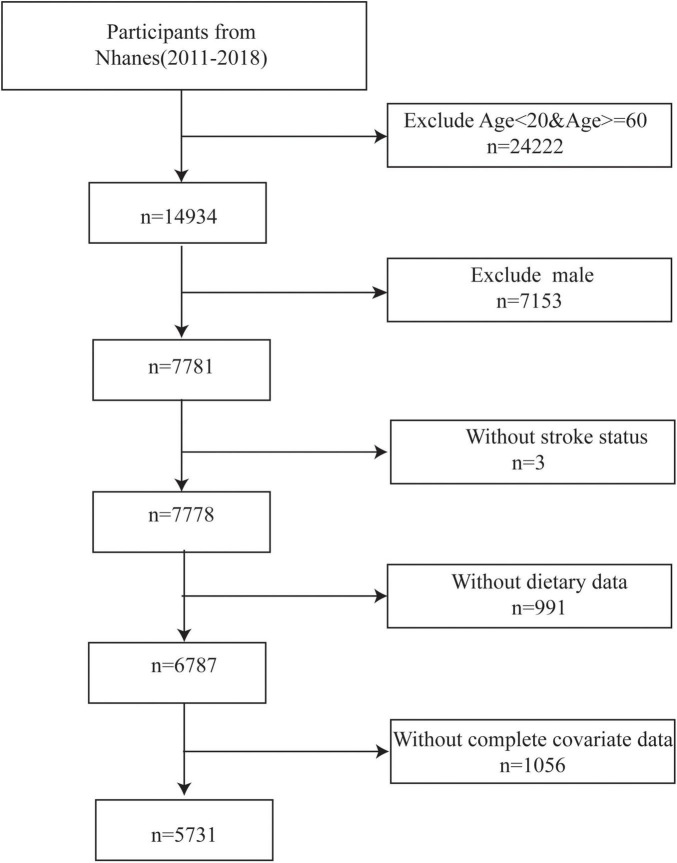
Flowchart.

The data used in the present study obtained from the National Health and Nutrition Survey (NHANES), which is designed to investigate the country’s health and nutrition status created by the Centers for Disease Control and Prevention (CDC) in America (43). And it is conducted in a 2-year cycle. Data from surveys, laboratory results, physical examination results, food and nutritional supplement information, and demographic data were all gathered for the NHANES database. In the current study, we selected four NHANES cycles from 2011 to 2018. The inclusion criteria were as follows: (1) female, (2) between the ages of 20 and 60 years, (3) having complete stroke status (Those who clearly answered whether they had a stroke.), (4) participants with complete dietary data. Finally, this survey included 5731 participants (Figure 1).

### 2.2 Definition of stroke

The Medical Condition Questionnaire (MCQ) was intended to elicit personal stroke history. In the MCQ, participants were asked if they had ever received information about a previous stroke diagnosis from a medical provider. The participants were considered as having suffered a stroke with answering “yes.” Those who answered as unclear were excluded.

### 2.3 Tea consumption

All participants were permitted to get involved in two 24-h dietary recall interviews. The first recall interview was obtained in an NHANES Mobile Examination Center (MEC). After 3 to10 days, a second dietary recall interview was completed over the phone. The food or drink consumed by each participant had a unique code, and the occasion, time, weight and other relevant information of eating were recorded in detail. Components from each food/beverage were calculated by the data from Food and Nutrient Database provided by the United States Department of Agriculture (44). By querying the food codes provided by the NHANES, we determined whether the participants drank tea or not. In this study, tea consumption was averaged over the two recall periods (if just one day was available, the value was utilized rather than an average). First, we calculated the quartiles of tea consumption among the participants who drunk tea, and then divided these tea drinkers into four groups based on this. Finally, adding those who did not drink tea, all participants were divided into five groups: (1) 0 g/ day, (2) > 0& ≤ 307.5 g/day, (3) > 307.5& ≤ 480 g/day, (4) > 480& ≤ 744 g/day, (5) > 744 g/day.

### 2.4 Covariates

Referring to related studies (6, 45, 46), we also investigated some potential confounding factors. Demographic characteristics among which were age, race, family income (poverty income ratio, PIR), level of education, smoking status, and drinking status were gathered through standardized questionnaires and in-person interviews. Body mass index (BMI) was from physical examination and is separated into three different parts based on the standard classification: (1) normal weight (18.5–24.9), (2) underweight (< 18.5), (3) overweight (≥ 25.0). In addition, we also investigated the history of three chronic diseases of the participants. Using the Diabetes Questionnaire (DIQ), we were able to diagnose diabetes. When participants confirmed an affirmative reply to the query “Did a doctor inform you of having diabetes?,” they were considered to have diabetes. The determination of hypertension was similar to aforementioned method, but the answer was based on the Blood Pressure and Cholesterol Questionnaire (BPQ). Besides those who were diagnosed as hyperlipidemia in self-reported diagnosis (based on the BPQ), participants considered as having hyperlipidemia as long as they met one of the following four conditions: (1) triglycerides ≥ 150 mg/dL, (2) total cholesterol ≥ 200 mg/dL, (3) low-density lipoprotein ≥ 130 mg/dL, (4) high-density lipoprotein ≤ 50 mg/dL (47).

### 2.5 Statistical analysis

Because of the sampling methodology employed in the survey, NHANES created various sample weights to use in the analysis. So, we integrated sample weights for dietary intake data associated with varying study periods into our analytical approaches. In accordance with the analytical guidelines of the NHANES (48), a new sample weight was devised by dividing the original 2-year sample weight by four when amalgamating four 2-year cycles of the ongoing data. All tests were two-sided, with a significance level of *P* < 0.05. All data analysis were conducted using R studio (version 4.4.0).^1^ The raw data can be found in the Supplementary Tables 2, 3. Similarly, the computation code was also provided in the Supplementary materials.

When analyzing the baseline data of stroke and non-stroke populations, the continuous variable uses *t*-test or non-parametric test, and the classification variable using cardinal test. We estimated the ORs and 95% confidence intervals (CIs) for the association between tea consumption and stroke using weighted multivariable-adjusted logistic regression models, including two adjusted models (Model II and Model III) and one non-adjusted model (Model I). Adjustments for age and race were performed in Model II, and Model III was further adjusted for PIR, educational level, smoke, alcohol use, BMI group, diabetes, hypertension and hyperlipidemia. We conducted this analysis by considering the amount of tea consumption as a continuous variable and a categorical variable, respectively. Subgroup analyses were carried out to investigate the existence of significant interactions between the link between tea consumption and stroke and several factors, including race, educational level, BMI group, smoke, alcohol use, diabetes, hypertension, and hyperlipidemia. Similarly, association between sugar added and stroke was also analyzed using the aforementioned two methods.

Taking into account the special group of women, we included menopause status as a covariate to conduct a sensitivity analysis. This data was derived from the Nhanes’s survey questionnaire that focused on females’ reproductive health in particular. Those who answered the age at last menstrual period are considered to be menopausal.

## 3 Results

### 3.1 Baseline characteristics

According to the results, the incidence of stroke in adult females was 1.74% in this study (100/5371). And approximately 37.47% participants have the habit of drinking tea. Table 1 contains detailed baseline information for each participant. Overall, stroke risk was substantially associated with age (*P* < 0.0001), race (*P* = 0.0159), education (*P* = 0.005), income (*P* < 0.0001), smoke (*P* = 0.0004), hypertension (*P* < 0.0001), diabetes (*P* < 0.0001), and hyperlipidemia (*P* = 0.0001). Stroke patients were older, less educated, had lower income, and had a greater rate of hypertension, diabetes, and hyperlipidemia than non-stroke individuals. However, stroke was not associated with alcohol use or BMI according to this study.

**TABLE 1:** Baseline characteristics, weighted.

Characteristic	Overall (*n* = 5731)	No stroke (*n* = 5631)	Stroke (*n* = 100)	*P*
**Age (years)**	39.91 (39.26, 40.55)	39.79 (39.15, 40.42)	47.91 (44.17, 51.65)	**<0.0001**
**Race (%)**				0.0159
Non-Hispanic White	61.93 (57.91, 65.79)	61.94 (57.92, 65.80)	61.16 (46.30, 74.20)	
Non-Hispanic Black	12.46 (10.43, 14.83)	12.30 (10.31, 14.61)	23.28 (14.52, 35.15)	
Mexican American	9.50 (7.61, 11.80)	9.54 (7.63, 11.86)	6.99 (2.61, 17.42)	
Other	16.11 (14.51, 17.86)	16.23 (14.61, 17.99)	8.57 (4.10, 17.07)	
**Education (%)**				0.0005
Less than High school graduate	10.70 (9.33, 12.26)	10.63 (9.24, 12.19)	15.80 (9.70, 24.68)	
High school graduate	20.08 (18.06, 22.27)	19.82 (17.75, 22.07)	37.27 (25.32, 51.01)	
Above High school graduate	69.21 (66.15, 72.11)	69.55 (66.44, 72.49)	46.93 (32.96, 61.40)	
**PIR**	2.87 (2.77, 2.97)	2.89 (2.78, 2.99)	1.86 (1.43, 2.28)	<0.0001
**Alcohol use (%)**				0.8682
No	21.08 (19.03, 23.30)	21.07 (18.98, 23.31)	22.13 (11.63, 38.03)	
Yes	78.92 (76.70, 80.97)	78.93 (76.69, 81.02)	77.87 (61.97, 88.37)	
**Smoke (%)**				0.0004
No	79.68 (77.73, 81.50)	79.94 (78.03, 81.72)	62.92 (50.10, 74.14)	
Yes	20.32 (18.50, 22.27)	20.06 (18.28, 21.97)	37.08 (25.86, 49.90)	
**BMI (kg/m^2^)**	29.59 (29.18, 30.00)	29.56 (29.14, 29.98)	31.91 (29.37, 34.44)	0.0860
**BMI GROUP (%)**				0.2961
normal weight	31.75 (29.63, 33.96)	31.92 (29.71, 34.20)	21.25 (10.53, 38.21)	
underweight	2.30 (1.85, 2.86)	2.28 (1.82, 2.85)	4.00 (0.94, 15.53)	
overweight	65.94 (63.54, 68.27)	65.81 (63.31, 68.22)	74.75 (57.74, 86.51)	
**Hypertension (%)**				<0.0001
No	78.50 (76.76, 80.15)	79.12 (77.38, 80.75)	38.28 (26.22, 51.98)	
Yes	21.50 (19.85, 23.24)	20.88 (19.25, 22.62)	61.72 (48.02, 73.78)	
**Diabetes (%)**				<0.0001
No	93.44 (92.44, 94.31)	93.60 (92.59, 94.48)	82.91 (76.07, 88.10)	
Yes	6.56 (5.69, 7.56)	6.40 (5.52, 7.41)	17.09 (11.90, 23.93)	
**Hyperlipidemia (%)**				0.0001
No	44.39 (41.87, 46.93)	44.76 (42.20, 47.34)	20.26 (11.66, 32.86)	
Yes	55.61 (53.07, 58.13)	55.24 (52.66, 57.80)	79.74 (67.14, 88.34)	
**Tea consumption (g/day)**	224.12 (203.97, 244.27)	2.26 (2.05, 2.46)	1.31 (0.63, 1.98)	0.0098
**Tea** **consumption groups** (**%)**				0.0156
0g/day	62.53 (60.09, 64.91)	62.29 (59.85, 64.67)	78.13 (66.86, 86.35)	
0-307.5g/day	9.55 (8.40, 10.84)	9.56 (8.41, 10.85)	8.87 (4.21, 17.71)	
307.5-480g/day	9.58 (8.50, 10.79)	9.68 (8.58, 10.91)	2.82 (0.98, 7.84)	
480&-744g/day	9.28 (8.34, 10.33)	9.35 (8.40, 10.40)	4.93 (1.98, 11.78)	
> 744g/day	9.06 (7.73, 10.58)	9.11 (7.78, 10.65)	5.25 (1.93, 13.50)	
**Sugary (%)**				0.0026
non-tea drinkers	62.53 (60.09, 64.91)	62.29 (59.85, 64.67)	78.13 (66.86, 86.35)	
tea without sugar	18.22 (16.25, 20.37)	18.40 (16.42, 20.56)	6.38 (2.81, 13.83)	
tea with sugar	19.25 (17.39, 21.26)	19.31 (17.45, 21.31)	15.49 (9.02, 25.30)	

PIR, poverty income ratio; BMI, Body Mass Index. Weighted mean (95% CI) was used to present continuous variables, while proportions (95% CI) were used to represent categorical variables.

### 3.2 The association between tea consumption and stroke

This study used three models to investigate the association between tea consumption and stroke. Firstly, we analyzed the tea consumption as a continuous variable and converted the unit to 100 g/day. Our analysis showed no correlation between tea consumption and stroke in model I, but after mixed-factor adjustment, the results showed that tea consumption was associated with a negative risk of stroke. Model II demonstrated that for every 100 g of tea consumed, the likelihood of stroke decreased by 10% (OR = 0.90, 95%CI: 0.81–0.99). In the model III that adjusted all covariates, the risk was reduced by 9% (OR = 0.91, 95%CI: 0.83–1.00). Then categorized tea consumption were analyzed in the linear regression. Results demonstrated that, in comparison to those who did not consume tea, those who drank 307.5–480 g of tea one day had a considerably decreased risk of stroke in the crude model (OR = 0.23, 95%CI: 0.08–0.64). This association persisted even after adjustment (Model II: OR = 0.23, 95%CI: 0.08–0.64; Model III: OR = 0.23, 95%CI:0.08-0.66). In Models II and III, consuming 480-744 g of tea per day lowered participants’ risk of stroke, and this associate was statistically significant (Model II: OR = 0.39, 95%CI 0.16–0.94; Model III: OR = 0.42, 95%CI: 0.18–0.98). In our study, however, consuming lower tea (≤ 307.5 g/day) did not appear be linked to stroke. Finally, results from our indicated that there was significant relationship between tea consumption and stroke across all the tea consumption categories by trend test (Model I *P* for tend = 0.0063, Model II *P* for tend = 0.0021, Model III *P* for tend = 0.0026). All the above results were shown in Table 2.

**TABLE 2:** Association between tea consumption and stroke, weighted.

Exposure	Model I OR (95%CI)	*P*	Model II OR (95%CI)	*P*	Model III OR (95%CI)	*P*
Tea Consumption (100g/day)	0.92 (0.83, 1.01)	0.069	0.90 (0.81, 0.99)	0.035	0.91 (0.83, 1.00)	0.041
**Tea Consumption Groups (g/day)**						
< = 0	Ref		Ref		Ref	
>0, < = 307.5	0.79 (0.36, 1.74)	0.6	0.70 (0.32, 1.55)	0.4	0.89 (0.38, 2.08)	0.8
>307.5, < = 480	0.23 (0.08, 0.64)	0.006	0.23 (0.08, 0.64)	0.006	0.23 (0.08, 0.66)	0.007
>480, < = 744	0.44 (0.19, 1.05)	0.064	0.39 (0.16, 0.94)	0.037	0.42 (0.18, 0.98)	0.045
>744	0.43 (0.15, 1.18)	0.10	0.35 (0.12, 0.98)	0.046	0.36 (0.12, 1.06)	0.036
*P* for trend	0.0063		0.0021		0.0026	

Model I: no adjustment. Model II: adjust for age, race. Model III: adjust for age, race, PIR, educational level, smoke, alcohol use, BMI group, diabetes, hypertension and hyperlipidemia.

### 3.3 Subgroup analysis for the association between tea consumption and stroke

**FIGURE 2:**
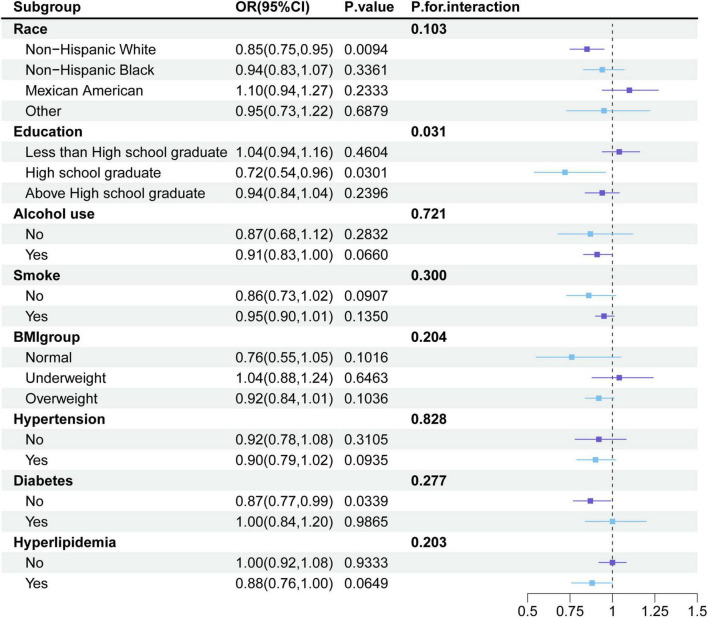
Subgroup analysis for the association between tea consumption and stroke, weighted.

Furthermore, we conducted detailed subgroup analyses. As illustrated in Figure 2, the associations between tea consumption and stroke did not significantly differ among race groups, BMI groups, smoke groups, alcohol use groups, hypertension groups, diabetes groups and hyperlipidemia groups. At the same time, we also found that the association between tea consumption and stroke also existed in white people (OR = 0.85, 95%CI: 0.75–0.85), people with high school education (OR = 0.72, 95%CI: 0.54–0.96) and people without diabetes (OR = 0.87, 95% CI: 0.77–0.99). However, we found tea may benefit the participants with a high school education more compared to those with other levels of education (*P* for interaction = 0.031).

### 3.4 The association between sugar added and stroke

Table 1 showed whether sugar in tea is connected with the incidence of stroke (*P* = 0.026) and the lower incidence (6.38%) among non-sugar tea consumers. Thus, the participants were divided into three groups:(1) non-tea drinkers (2) tea without sugar (3) tea with sugar. Then, we used weighted multivariable-adjusted logistic regression models to examine whether there was sugar in tea would affect the risk of stroke (shown in the Table 3). Compared to those who did not drink tea, participants who drank non-sugar tea had a reduced chance of stroke (Model I: OR = 0.28, 95%CI: 0.12–0.64; Model II: OR = 0.23, 95%CI: 0.10–0.56; Model III: OR = 0.28, 95%CI: 0.11–0.69). It was worth emphasizing that drinking sugared tea had no association to stroke in all models (Model I: OR = 0.64, 95%CI: 0.64–1.15; Model II: OR = 0.59, 95%CI: 0.32–1.06; Model III: OR = 0.59, 95%CI: 0.34–1.02).

**TABLE 3:** Association between sugar added and stroke, weighted.

Exposure	Model I OR (95%CI)	*P*	Model II OR (95%CI)	*P*	Model III OR (95%CI)	*P*
Non-tea drinkers	Ref		Ref		Ref	
Tea without sugar	0.28 (0.12, 0.64)	0.003	0.23 (0.10, 0.56)	0.002	0.28 (0.11, 0.69)	0.07
Tea with sugar	0.64 (0.64, 1.15)	0.13	0.59 (0.32, 1.06)	0.076	0.59 (0.34, 1.02)	0.06

Model I: no adjustment. Model II: adjust for age, race. Model III: adjust for age, race, PIR, educational level, smoke, alcohol use, BMI group, diabetes, hypertension and hyperlipidemia.

### 3.5 Subgroup analysis for the association between sugar added and stroke

Finally, we conducted detailed subgroup analyses to analyze the association between sugar added and stroke. In each subgroup, participants who did not drink tea were taken as control. Due to the insufficient sample size in the subgroup, there were results with an OR of 0 (Mexican American/Less than High school graduate) as well as instances where the OR value could not be calculated (BMI group). As shown in Table 4, there were no significant differences in the association of sugar added and stroke across all different groups (*P* for interaction > 0.05). However, data indicates that certain categories of people are more likely to benefit from sugar-free tea, lowering their risk of stroke. However, the results indicated that certain categories of people were more likely to benefit from sugar-free tea, lowering their risk of stroke, such as non-Hispanic White (OR = 0.16; 95%CI: 0.03.–0.79), Above High school graduate (OR = 0.33; 95%CI: 0.12–0.95), those without alcohol using (OR = 0.13; 95%CI: 0.02–0.80), those with alcohol using (OR = 0.33; 95%CI 0.11–0.94), those didn’t smoke (OR = 0.26; 95%CI: 0.09–0.74), those with Hypertension (OR = 0.16; 95%CI: 0.05–0.22), those without Diabetes (OR = 0.28; 95%CI: 0.11–0.70), those with Hyperlipidemia (OR = 0.27; 95%CI: 0.09–0.77). Although some subgroup results showed that drinking tea with sugar was negatively correlated with stroke, the OR was greater than that of the group drinking tea without sugar. Interestingly, we also found some inconsistent results, where only the impact of sugary tea on stroke was statistically significant among individuals with a high school education (OR = 0.12; 95%CI: 0.02–0.91).

**TABLE 4:** Subgroup analysis for the association between sugar added and stroke, weighted.

Subgroup	OR (95%CI)	*P*-value	Interaction *P*-value
**Race**			0.081
**Non-Hispanic White**			
Tea without sugar	0.16 (0.03, 0.79)	0.025	
Tea with sugar	0.32 (0.17, 0.59)	0.001	
**Non-Hispanic Black**			
Tea without sugar	1.29 (0.48, 3.48)	0.6	
Tea with sugar	1.21 (0.45, 3.28)	0.7	
**Mexican American**			
Tea without sugar	0 (0, 0)	<0.001	
Tea with sugar	3.24 (0.54, 1.96)	0.2	
**Other**			
Tea without sugar	0.24 (0.02, 2.66)	0.2	
Tea with sugar	0.55 (0.05, 5.86)	0.6	
**Education**			0.070
**Less than High school graduate**			
Tea without sugar	0 (0, 0)	<0.001	
Tea with sugar	1.58 (0.54, 4.65)	0.4	
**High school graduate**			
Tea without sugar	0.24 (0.05, 1.13)	0.07	
Tea with sugar	0.12 (0.02, 0.91)	0.041	
**Above High school graduate**			
Tea without sugar	0.33 (0.12, 0.95)	0.04	
Tea with sugar	0.84 (0.38, 1.84)	0.6	
**Alcohol use**			0.718
**No**			
Tea without sugar	0.13 (0.02, 0.80)	0.029	
Tea with sugar	0.47 (0.10, 2.16)	0.3	
**Yes**			
Tea without sugar	0.33 (0.11, 0.94)	0.038	
Tea with sugar	0.65 (0.36, 1.17)	0.15	
**Smoke**			0.224
**No**			
Tea without sugar	0.26 (0.09, 0.74)	0.013	
Tea with sugar	0.38 (0.15, 0.94)	0.037	
**Yes**			
Tea without sugar	0.18 (0.02, 1.77)	0.14	
Tea with sugar	1.13 (0.56, 2.25)	0.7	
**Hypertension**			0.248
**No**			
Tea without sugar	0.5 (0.11, 2.24)	0.4	
Tea with sugar	0.33 (0.10, 1.10)	0.07	
**Yes**			
Tea without sugar	0.16 (0.05, 0.55)	0.005	
Tea with sugar	0.74 (0.35, 1.59)	0.4	
**Diabetes**			0.293
**No**			
Tea without sugar	0.28 (0.11, 0.70)	0.008	
Tea with sugar	0.47 (0.22, 0.99)	0.046	
**Yes**			
Tea without sugar	0.43 (0.06, 3.08)	0.4	
Tea with sugar	1.57 (0.47, 5.17)	0.5	
**Hyperlipidemia**			0.119
**No**			
Tea without sugar	0.39 (0.10, 1.57)	0.2	
Tea with sugar	1.93 (0.56, 6.67)	0.3	
**Yes**			
Tea without sugar	0.27 (0.09, 0.77)	0.016	
Tea with sugar	0.43 (0.19, 0.98)	0.045	

### 3.6 Sensitivity analysis

We have reconsidered the relationship between stroke and tea consumption by incorporating menstrual status into three separate models. The result was the same as before (shown as Supplementary Table 1). Furthermore, we calculated the interaction among the three factors, and the result showed that menstrual status did not affect the association between stroke and tea consumption (*P* for interaction = 0.713).

## 4 Discussion

This study which involved 5,731 adult females under 60 explored whether tea was linked to stroke by analyzing data from the 2011–2018 NHANES. First, we discovered that increasing tea drinking lowers the risk of stroke in an adult female by 9% for every 100 grams eaten per day. The risk of stroke is lowest when tea consumption is between 307.5 and 480 g/day (OR = 0.23), according to an analysis of tea consumption based on categorized characteristics. Furthermore, we discovered that consuming tea without added sugar decreased the incidence of stroke; however, this association was not present in participants who drank tea with added sugar. In summary, this study suggested that drinking tea can be used as a healthy diet to prevent strokes in adult females.

It is widely acknowledged that good diet is one of the simplest and most effective approaches to prevent stroke. According to studies, multiple dietary habits have been shown to prevent stroke, such as Mediterranean Diet (49, 50), which also recommends drinking tea (51, 52). With tea being one of the world’s most popular beverages, its association with the incidence of stroke has been researched. Drinking two to three cups (1 cup = 236 ml) of tea a day could reduce the incidence of stroke by thirty-two percent, according to a cohort research conducted at the UK Biobank (HR = 0.68, 95%CI: 0.59–0.79; *P* < 0.001) (53). Seung-Mi Lee et al. (54) in South Korea conducted a multi-center case-control study to investigate the link between drinking tea and hemorrhagic stroke, and the findings revealed that drinking green tea can prevent hemorrhagic stroke. Furthermore, a meta-analysis involving 645,393 individuals showed that tea consumption decreases the possibility of stroke, particularly hemorrhagic stroke (22). At the same time, when we went through the literature, we discovered that there was not much information on stroke preventive strategies specific to females, so we need to explain the association between stroke and tea consumption, particularly among adult females in the US. On the basis of the data of adult females from NHANES, our study confirmed the existence of this association and showed that drinking tea in moderation lowered the risk of stroke, which is consistent with the findings of previous studies. In our study, we also classified the amount of tea consumption, and the results showed that an adverse association was found in all three models only when tea consumption ranged between 307.5 g and 480 g per day, implying that moderate tea consumption benefits prevention. Previous research has also indicated that moderate tea consumption is the most appropriate. A previous meta-analysis of cohort studies (22) found that moderate green tea consumption can reduce the risk of stroke (When the daily tea consumption was 500 ml, the relative risk (RR) value was the smallest). A study in the UK found that individuals who consume 2–3 cups of tea daily have the lowest risk of mortality compared to those who drink other amounts of tea (55). Furthermore, the greatest reduction in ischemic stroke risk was associated with consuming one to two cups of green or oolong tea each day, which was found in a case-control study (56). Although the optimal amount of tea consumption for the lowest stroke risk varied in these reports, they all indicated that more was not necessarily better for preventing stroke. Our results were consistent with these, and we had identified the optimal tea consumption (307.5–480 g/day) amount for preventing stroke in American adult females (20–60 years). Considering the impact of menopause on stroke, we took menopause status into account in the sensitivity analysis, but the association between tea consumption and stroke still existed, which further indicated that our results were robust. After doing more analysis on the data, we discovered that drinking tea without added sugar was more likely to lower the risk of stroke, which was our new insight. The result was consistent with the current dietary recommendation to restrict sugar intake in moderation, which was not addressed in the tea study that was conducted.

Only 1.74% people in this study suffered from stroke, which may be related to the small number of people involved and the defects of the definition of stroke. In the analysis of the baseline data, this study found no difference in the distribution of the number of drinkers and BMI between stroke and non-stroke groups. In the analysis of the baseline data, this study found no difference in the distribution of the number of drinkers and BMI between persons who had a stroke and those who did not. These results are inconsistent with previous research (53), potentially due to the limited sample size in this study. Furthermore, the only criterion for classifying participants’ alcohol consumption in this study was whether they consumed alcohol. However, recent studies have shown that moderate alcohol consumption may actually reduce the risk of stroke, so the current results may be due to the lack of more detailed classification (54). But this result was also compatible with those of a case-control research on strokes in young adults (57). Regarding BMI, it is noteworthy that most participants in this study had a high BMI, which may lead to a certain degree of bias in the data.

In the stratified study, the association between tea consumption and stroke was not significant in all subgroups, which may be related to the reduction in sample size in each subgroup. However, the negative association between stroke and tea drinking was present among white participants, those with the highest level of education (high school), and those without diabetes. In addition, we also performed subgroup analysis to examine the association between sugar added and stroke. The interaction test results indicated that the relationship between stroke and tea consumption was not influenced by these groupings. But the results suggested that a more specific group of individuals could have prevented stroke by drinking tea without sugar, such as non-Hispanic White, above High school graduate, those without alcohol using, those with alcohol using, those didn’t smoke, those with Hypertension, those without Diabetes and those with Hyperlipidemia. These results provide evidence for public health departments to develop specific stroke prevention programs for these certain demographic groups. For sure, high-risk individuals, those with Hypertension or Hyperlipidemia, could also receive more detailed prevention methods, such as drinking tea without sugar.

Fertility and menopausal transition are processes that women in this age group (20–60 years) will experience, and these experiences are related to stroke. Therefore, we attempt to explain the relationship between tea consumption and stroke by looking at the effects of tea on these processes. First, we posit that tea may help alleviate the pro-inflammatory impacts resulting from unstable estrogen secretion in females’ physiology. Estrogen regulates a variety of physiological processes in the female body. Although estrogen is believed to be beneficial to the human body and exerts protective effects on stroke through various pathways such as anti-inflammation, anti-oxidative, inhibition of platelet agglutination, and promotion of nerve growth factor expression (58), changes in hormone levels due to menarche, menopause, pregnancy, childbirth, and miscarriage can easily lead to a weakening or alteration of these effects. Studies indicate that reduced estrogen secretion and the diminishing of its protective benefits are closely linked to a heightened susceptibility to strokes, particularly among perimenopausal (59) and postmenopausal females (60, 61). The Global Burden of Disease Study 2019 (62) estimated that, globally, 14.8% of females of childbearing age used exogenous estrogen as a form of contraception, compared to 21.8% in the US. Meanwhile, exogenous estrogens (hormone replacement therapy) are frequently employed in the management of female health issues such as amenorrhea, infertility, and migraines. But epidemiological surveys showed that females using exogenous estrogen were more likely to experience stroke (63–65), as exogenous estrogen had pro-inflammatory effects (66, 67). On the other hand, persistent inflammatory reactions are essential for the onset of stroke, as a result, the higher risk of stroke in females could be attributed to lower anti-inflammatory and vascular protective effects generated by estrogen alterations. Although studies have shown that tea can increase estrogen levels in females (68), the anti-inflammatory effects of tea should not be underestimated. A cross-sectional study involving adult females aged 18–48 has shown that tea can reduce inflammatory factors in the body, thus playing an anti-inflammatory role (69). Tea’s functional components, including tea polyphenols (70), l-theanine (71), theaflavin (72), and caffeine (73), have been proven to lower the expression of inflammatory factors (74–76) such as IL-6, IL-1, and TNF-α, leading to anti-inflammatory effects. There were the same results in animal experiments. An experiment on female rats showed that green tea polyphenols could decrease the levels of white blood cells in the body, thereby suppressing the inflammatory response in the body (77). Thus, we assumed that tea’s anti-inflammatory properties contribute to a lower risk of stroke.

Second, we believe that tea’s antithrombosis may explain its association with stroke. According to reports, the incidence of stroke associated with pregnancy and childbirth is about 0.03% (78). Special physiological changes during pregnancy are intimately associated to thrombosis, increasing the risk of stroke in females (79). Increased blood coagulation and fibrinogen levels during pregnancy cause pregnant females to enter a hypercoagulable state (80) and are susceptible to prethrombotic state (PTS). PTS is a pathological dysfunction or disturbance of the coagulation, anticoagulation, and fibrinolysis systems in the human body caused by a variety of factors, and it also increases the risk of stroke (81). Another study showed that females with recurrent fetal loss had a 78% chance of developing PTS after controlling for all other characteristics (82). Furthermore, estrogen-containing contraceptives may boost platelet coagulation, resulting in a rise in fibrinogen, which promotes blood clot formation (83). In short, females’ unique physiological reactions make them more susceptible to thrombosis than males, which is a key pathogenic mechanism in the stroke process, and drinking tea can help minimize this risk. An animal study discovered that theaflavin can suppress platelet activation in arterial clot model mice, as well as fibrinogen binding, and recommended that it be a novel food-based inhibitor of thrombotic disorders (84). The effect of tea polyphenols on inhibiting thrombosis has been confirmed (85). The similar conclusion has also been drawn from clinical randomized controlled studies. A Chinese clinical investigation discovered that individuals with high blood viscosity experienced a significant change in their hemorheology and a return to normal in the associated indicators following a 6-week yerba mate tea consumption (86). To put it briefly, tea has the ability to disrupt the production of blood clots, which lowers the risk of stroke in females.

Lastly, we suggest that tea could correct metabolic abnormalities, which reduces the possibility of suffering from strokes. As is generally known, metabolic abnormalities are an independent risk factor for stroke, and an 11.9-year study (87) discovered that those with metabolic anomalies had a 30% higher risk of stroke than those without metabolic problems (OR = 1.30, 95% CI: 1.09–1.56). In the current study, 69.54% of the subjects were overweight, and more than half of them had hyperlipidemia (Table 2), indicating that obesity and hyperlipidemia are more prevalent in females. In addition, females are more vulnerable to metabolic abnormalities during pregnancy, which can result in hyperlipidemia and diabetes. Increased oxidative stress and inflammation in the mother during pregnancy can increase insulin secretion function, triggering alterations in sugar metabolism (88); approximately one-sixth of pregnant females develop diabetes (89). During pregnancy, lipid metabolism changes, and pregnant females’ enhanced metabolism causes a slight increase in total blood cholesterol and triglyceride levels (90). Furthermore, during pregnancy, pregnant females tend to engage in less activity and consume more high-calorie foods, increasing their risk of metabolic abnormalities and obesity. Although these abnormalities go away after childbirth, females’ long-term health may be at risk if they develop into a pathological condition. It was demonstrated by a cross-sectional study that the number of pregnancies and live births are closely linked to metabolic syndrome, with more live births increasing the risk (OR = 1.18, 95% CI: 1.04–1.35) (91). Females in perimenopause, as well as those of childbearing age, are at the risk. A cohort study involving healthy females in perimenopausal period revealed that the decline in estrogen levels resulted in abdominal obesity, elevated body mass index, increased total cholesterol and low-density lipoprotein levels, decreased high-density lipoprotein levels, heightened fasting blood glucose, and elevated blood pressure (92), all of which increase the chance of a stroke. The tea can just alleviate these problems. A literature review documented that tea could affect females’ blood glucose and insulin resistance, playing a beneficial role in their metabolism (93). A clinical randomized controlled trial specifically targeting females has shown that green tea extract (Epigallocatechin gallate) can not only aid in weight loss but also reduce total cholesterol levels as well as LDL-C (94). Concurrently, a prospective study discovered that tea consumption can successfully lower the risk of stroke in individuals with central obesity (OR = 0.60, 95% CI: 0.44–0.81) (95). This finding offers trustworthy proof that tea can lower the risk of disease in high-risk populations, including those who are obese or have metabolic abnormalities. In conclusion, the impact of tea on lowering blood lipid and blood glucose can elucidate its contribution to mitigating the risk of stroke in adult females.

There are still some limits to our study. First of all, the statistical data on tea consumption was calculated by the participants’ memories, which could introduce recall bias and may not precisely represent the participants’ true tea consumption. Furthermore, the amount of tea consumed on only two days may not be a reliable indicator of the participants’ long-term dietary habits. However, it has also been proposed that this method can be a valid representation of their daily dietary intake (96). Secondly, the inclusion of stroke and several chronic conditions among the covariates was dependent on the participants’ own responses, which might be prone to recall bias, likewise, some participants may lack a good awareness of their health circumstances, which could influence the results. Thirdly, several factors increase the risk of stroke, including genetics, medicine, and physical activity. Even if we included certain confounding factors in our analysis, there could still be residual confounding. Fourthly, various varieties of tea, including oolong, green, and black, can impact stroke risk (54). Furthermore, the temperature of tea (hot or iced) and other ingredients used in tea (milk or juice) can affect the results of research (97). However, our current study did not distinguish between these elements, emphasizing the necessity for more detailed subgroup analysis in future investigations. Finally, given that the study was cross-sectional, the causal association between stroke and tea consumption was not clear. More randomized controlled trials and animal experiments are required to ascertain the association between tea consumption and the occurrence of stroke in females.

## 5 Conclusion

In this study based on the NAHNES database, there was an association between tea consumption and stroke. Our study demonstrated that moderate tea consumption (307.5–480 g/day) can reduce the risk of stroke in American adult females (20–60 years). Furthermore, our findings suggest that tea without sugar is more beneficial to female. This study can serve as a reference for formulating preventive measures against stroke for American adult females. In the future, healthcare professionals may include a daily moderate intake of tea (307.5–480 g/day) as part of a healthy diet to help prevent stroke.

## Data Availability

The original contributions presented in the study are included in the article/Supplementary material, further inquiries can be directed to the corresponding author.

## Footnotes

1
https://www.r-project.org
