# Supplementary material for: Association between tea consumption and stroke in the American adult females: analyses of NHANES 2011–2018 data
Source: Front Nutr. 2024 Oct 22;11:1452137. doi: 10.3389/fnut.2024.1452137 (PMC11534590; doi:10.3389/fnut.2024.1452137)
Supplement: Supplementary file 1 [file Table_1.docx]

Table S1 Association between tea consumption and stroke(adjust for menstrual status),Weighted

| **Exposure** | **Model I**  **OR(95%CI)** | **P** | **Model** **II**  **OR(95%CI)** | **P** | **Model** **Ⅲ**  **OR(95%CI)** | **P** |
| --- | --- | --- | --- | --- | --- | --- |
| Tea consumption(100g/day) | 0.90(0.82, 0.99) | 0.033 | 0.90(0.81, 0.99) | 0.035 | 0.91(0.83, 1.00) | 0.041 |
| Tea consumption groups(g/day) |  |  |  |  |  |  |
| <=0 | Ref |  | Ref |  | Ref |  |
| >0, <=307.5 | 0.73(0.34, 1.60) | 0.4 | 0.68(0.32, 1.47) | 0.3 | 0.80(0.34, 1.85) | 0.6 |
| >307.5, <=480 | 0.24(0.08, 0.66) | 0.007 | 0.23(0.08, 0.64) | 0.006 | 0.25(0.09, 0.71) | 0.010 |
| >480, <=744 | 0.38(0.15,0.96) | 0.040 | 0.37( 0.15,0.92) | 0.034 | 0.38(0.16,0.95) | 0.038 |
| >744 | 0.37(0.13, 1.03) | 0.057 | 0.37(0.13, 1.07) | 0.065 | 0.39(0.13, 1.15) | 0.086 |
| P for trend | 0.0020 |  | 0.0021 |  | 0.0026 |  |

Model I: adjust for menstrual status.

Model II: adjust for menstrual status,age, race.

Model Ⅲ: adjust for menstrual status,age, race, PIR, educational level, smoke, alcohol use, BMI group, diabetes, hypertension and hyperlipidemia.
